# Orbital Pseudotumor: Distinct Diagnostic Features and Management

**DOI:** 10.4103/0974-9233.53370

**Published:** 2008

**Authors:** Imtiaz A Chaudhry, Farrukh A Shamsi, Yonca O Arat, Fenwick C Riley

**Affiliations:** 1 From Oculoplastic and Orbit Division, King Khaled Eye Specialist Hospital, Riyadh, Kingdom of Saudi Arabia; 2 From Research Department, King Khaled Eye Specialist Hospital, Riyadh, Kingdom of Saudi Arabia; 3 From Department of Ophthalmology, Baylor College of Medicine, Houston, TX 77030, United States of America; 4 From Pathology Department, King Khaled Eye Specialist Hospital, Riyadh, Saudi Arabia

**Keywords:** orbital pseudotumor, inflammation, diagnosis, management

## Abstract

**Purpose:**

To provide an overview of the spectrum of diseases known as ‘idiopathic orbital inflammatory syndrome’ also known as orbital pseudotumor, with emphasis on specific diagnostic challenges in the evaluation and management of patients with this disorder.

**Methods:**

Review of the relevant literature and summarize recent findings regarding the epidemiology, diagnosis, pathophysiology and treatment of orbital pseudotumor.

**Results:**

Orbital pseudotumor is a benign intraorbital process confined to the orbit but extra orbital involvement can occur. It is among the 3^rd^ most common orbital diseases along with thyroid orbitopathy and lymphoproliferative disorder and accounts for 5-10% of orbital processes. Clinically, orbital pseudotumor has been categorized as myositis, dacryoadenitis, anterior, apical and diffuse process. Patients may present with diplopia, conjunctival chemosis, proptosis or abnormal computed tomography scan (CT-scan) findings. Patients may also have associated optic neuropathy. Diagnosis is based on careful history, ultrasonography (U/S), CT-scan and magnetic resonance imaging (MRI) studies which may also provide prognostic information. Treatment consists of systemic corticosteroids in the form of oral or intravenous administration. Confirmation is made by orbital biopsy. In addition to radiation, cytotoxic agents, immunosuppressant, IV immunoglobulin, biological therapy, TNF-alpha inhibitor monoclonal antibody and Mycophenolate Moftil have been found to be useful in the management of refractory orbital pseudotumor.

**Conclusion:**

Understanding of the clinical features of patients with orbital pseudotumor, differentiating it from other orbital processes by use of imaging techniques and timely implementation of available treatment strategies may help prevent visual loss and associated morbidity from this condition.

First described by Birch-Hirschfield in 1905, ‘idiopathic orbital inflammatory syndrome,’ also known as orbital pseudotumor, is a nonspecific, non-neoplastic inflammatory process of the orbit.^1^–^3^ After Graves' disease and lymphoproliferative disorders, orbital pseudotumor is the 3^rd^ most common ophthalmologic disease of the orbit and account for approximately 8-11% of all the orbital tumors.^4^ Among the 1264 patients referred to Wills Eye Hospital, Philadelphia, USA for a suspected orbital mass, inflammatory lesions accounted for 11% of the histopathologically proven lesions.^5^ From the 200 consecutive patients aged 60 years or older evaluated at the same hospital with an orbital tumor, pseudotumor was found in 19 (10%) of cases.^6^ Grave's ophthalmopathy, orbital pseudotumor and orbital infection can occasionally demonstrate overlapping clinical features. Orbital inflammation, particularly the fibrotic form, has been recognized to represent a distinct disease entity that may require aggressive intervention.^7^ The ocular manifestations of orbital pseudotumor may include periorbital edema, erythema, proptosis, ptosis, diplopia and pain with eye movements.^2^,^3^,^8^–^10^ Patients with fast progressive proptosis, decreased ocular motility and pain are the most frequent alarm signals when these patients are seen in the emergency setting (Fig 1).^11^ Orbital pseudotumor and lymphoid tumor show distinct clinical, morphologic, immunophenotypic and molecular genetic characteristics. More patients with lymphoid tumors may have palpable mass compared to patients with orbital pseudotumor whereas more patients with orbital pseudotumor may have swollen eyelid, conjunctival congestion, pain, retinal folds or hemorrhage and optic nerve atrophy than patients with lymphoid tumors. Almost one third of the patients with orbital pseudotumor may not be easily differentiated pathologically from lymphoid tumors and require immunophenotypic and molecular genetic analyses based on polyclonal or monoclonal proliferation of lymphocytes.^12^ The current concept of orbital pseudotumor defines it as an idiopathic tumorous inflammation made up of a pleomorphic inflammatory cellular response and a fibrovascular tissue reaction. Histopathologic analysis show a spectrum of granulomatous inflammation, admixed with nongranulomatous inflammation and fibrosis.^13^ Although prevalent in the adult population, the disorder is less common in pediatric population.^14^–^16^ Evaluation and management of patients with orbital pseudotumor is challenging and the importance of its inclusion in the differential diagnosis of orbital disorders is necessary.^5^,^17^,^18^

**Figure 1:**
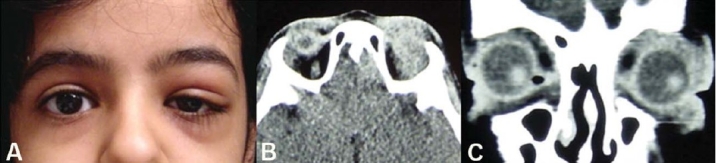
A 13 year girl presented with 1-month history of left upper eyelid swelling and pain (A). CT-scan (axial andcoronal) reveals inflammation of the left orbit (B and C).

The disease has been reported in all ethnic groups around the globe. Among the 209 cases reported from China (mean age 44.4 years; range 4-80 years), 90 had inflammation on the right side, 81 had on the left and 38 had both sides involvement.^12^ Proptosis was the most common presentation followed by swollen eyelids and motility restriction. According to radiologic and surgical findings focal mass within orbit was the most frequent subtype (43%), followed by lacrimal inflammatory pseudotumor (32%), diffuse orbital inflammation (10%) and myositis (8%). Less common findings were perineuritis (2%), periscleritis (2%), acute inflammation (2%) and eyelid pseudotumor (1%).^12^ Gunalp et al., 1996 reviewed their clinical experience with 132 orbital pseudotumors from Turkey and found proptosis in 82%, motility restriction in 54%, and visual acuity loss in 38% of the patients.^19^ Diffuse inflammatory process was the most frequent radiologic pattern followed by myositis and dacryoadenitis. Less common clinical features included focal encapsulated mass, Tolosa-Hunt syndrome, perineuritis and periscleritis.^19^ Orbital pseudotumor has also been reported in other animals including cats. In one report, six of the seven cats presented with unilateral orbital involvement that progressed to bilateral orbital disease despite treatment. Like humans, the onset has been found to be insidious, evolving over weeks to months. Histopathology of the affected orbital tissues in cats shows extensive fibrosis and encapsulation of normal tissues without characteristics of neoplasia. Clinical findings and histopathology of globes and orbital tissues in cats share many similarities to sclerosing orbital pseudotumor in humans. In cats, however, the prognosis for the eye globe appears to be poor.^20^ The cats may have progressive lagophthalmia, keratitis, and decreased ocular motility without any response to antibiotics and immunosuppressive agents. Exenteration of the eye may be required because of worsening clinical signs or corneal perforation due to orbital pseudotumor.^21^

## Pathogenesis

Pathogenesis of orbital pseudotumor remains elusive but several lines of evidence point to immune-mediated processes as the likely underlying ocular mechanism. The etiology of orbital pseudotumor is unknown, but infection, autoimmune disorder, and aberrant wound healings have been put forward as possibilities.^2^ The disorder has also been associated with infectious diseases such as Streptococcal pharyngitis, viral upper respiratory infection and Borrelia burgdorferi infection.^9^,^10^,^22^,^23^ Pathological findings may be non-specific and may only reveal benign lymphoid hyperplasia and inflammatory cell infiltration with necrotizing vasculitis.^24^ Biopsies obtained from orbital tissue may show complement deposits and increased expression of HLA class-I antigens in the intermuscular tissue. The biopsies from pseudotumor cases and Graves' ophthalmopathy cases contain increased numbers of intermuscular HLA class-II-expressing cells. In spite of clinical remission, the local condition in pseudotumor and Graves' ophthalmopathy suggest active inflammatory disease.^25^ Proliferating cell nuclear antigen (PCNA) activity in conjunction with the ratio of B-/T-cells may be a helpful immunohistologic adjunct for differentiating purely inflammatory lesions of the orbit from lymphoid tumors. In one study, PCNA activity was markedly increased in the higher grade lymphoma group compared to that in the low grade lymphoma and orbital pseudotumor group. Lymphoma cases may show a significantly increased B-/T-cell ratio compared to orbital pseudotumor.^26^,^27^ In addition, cANCA levels should be investigated in patients with orbital pseudotumor as a possible sign of Wegener's granulomatosis.^28^ Mimics of orbital pseudotumors include congenital orbital mass lesions or orbital neoplastic diseases such as lymphoma or rhabdomyosarcoma. One category of orbital pseudotumor may display a granulomatous inflammatory pattern that mimics sarcoidosis. The ophthalmologists should be aware of the existence of granulomatous orbital pseudotumors not associated with systemic sarcoidosis as a distinct clinicopathologic entity.^27^ Sarcoidosis may be revealed by an orbital inflammatory lesion. Steroids are necessary to prevent ocular functional complications.^29^

## Diagnosis

**Figure 2:**
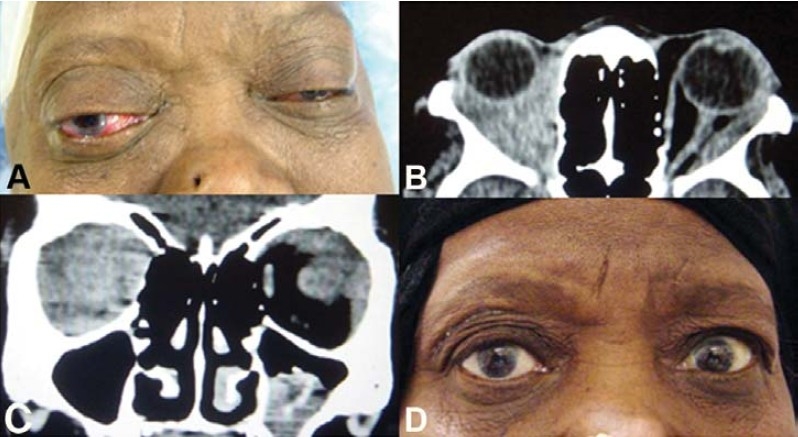
External photograph of a 60-year-old woman withhistory of several episodes ofrecurrent bilateral chemosis, restriction of extraocular motility and vision (A). CT-scan (axial and coronal) revealed bilateral infiltrative processes (B and C).Treatment with a course of corticosteroids and radiationtherapy resulted in resolution ofher symptoms (D).

**Figure 3:**
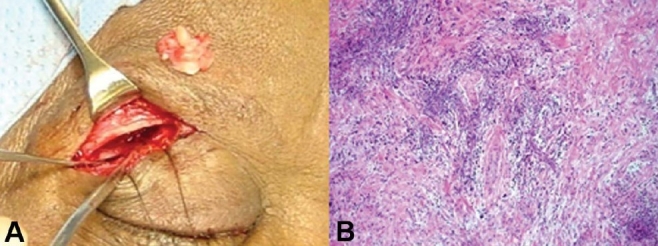
A biopsy specimen taken from right supra orbital region of patient in figure 2 (A), reveled granulo-matous inflammatory process (B).

Orbital pseudotumors are pathologic entities that often challenge ophthalmologists and radiologists. The imaging features of orbital lymphoma, leukemia and other lymphoproliferative disorders have been extensively studied by the use of MRI and CT-scan.^30^ On imaging studies, patients may show stigmata of orbital inflammation, including fluid in the Tenon's space, molting of the optic nerve contour and thickening of the extraocular muscles.^31^ On CT-scan and MRI studies, pseudotumors may present with diffuse orbital mass, uveoscleral thickening, contrast enhancement of Tenon's potential space, proptosis and optic nerve and extraocular muscle enlargement (Fig 2).^30^,^32^ Other helpful hints in orbital pseudotumor may include absence of contiguous paranasal sinus process usually noted in patients with orbital cellulites.^31^ On conventional MRI sequences subtle areas of inflammation or enhancing tissue can easily be masked by the high signal intensity of orbital fat and involvement of the fat itself may not be appreciated. Imaging features of patients with orbital pseudotumors have been studied by MRI using frequency-selective fat saturation and Gd-DTPA.^32^ Open biopsy is recommended for cases suspicious for an orbital malignancy or when a poor or equivocal response to corticosteroids is seen (Fig 3). The ultimate diagnosis and treatment plan relies on a careful history and detailed clinical examination followed by the judicious use of ancillary diagnostic testing and a comprehensive treatment plan.^9^

## Classification

**Figure 4:**
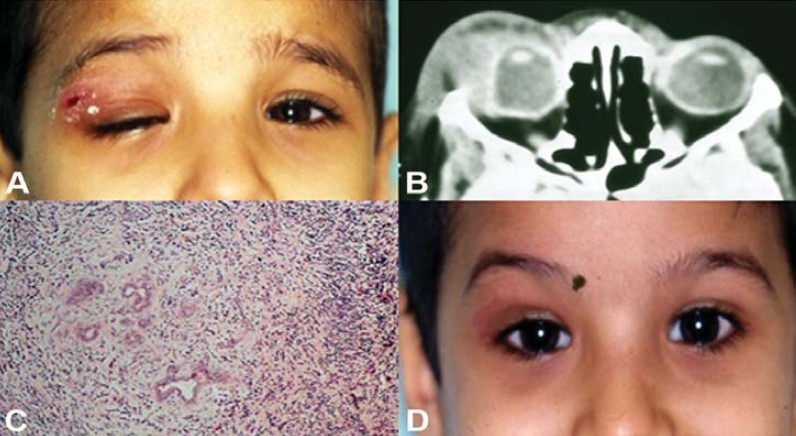
A 6 year old boypresented with ptosis and painfultender swelling over right uppereyelid (A). A CT-scan (axial)reveled enlarged lacrimal glandand associated swelling on theright side (B). Histopathology ofthe lacrimal gland reveledinflammatory infilteratesconsistant with dacryoadenitis(C). Course of oral corticoster-oids resulted in dramaticimprovement of his symptoms(D).

Orbital pseudotumor is often subclassified on the basis of the anatomic target areas within the orbit.^2^,^8^,^9^,^25^ Clinically, this may present primarily as a dacryoadenitis, a myositis, a trochleitis, an inflammatory process restricted to the vicinity of the superior orbital fissure and cavernous sinus (the Tolosa-Hunt Syndrome of painful ophthalmoplegia), or as a diffuse anterior soft-tissue inflammation (Fig 4).^9^,^10^,^33^,^34^ Unilateral periorbital pain, cranial nerve palsies and a dramatic response to corticosteroids therapy are the hallmarks of clinical presentation in Tolosa-Hunt-Syndrome and orbital pseudotumor. Apart from neuroradiological findings, almost similar histopathology and clinical presentation makes it difficult to distinguish between these two syndromes. Both are non-specific chronic granulomatous diseases of unknown origin, sharing significant clinical characteristics. Wasermeir et al., 2002 reported two patients suffering from acute granulomatous orbital pseudotumor who also fulfilled the criteria of Tolosa-Hunt-Syndrome.^34^ They suggested that both diseases belong to the same pathological process.^34^ A 10-year retrospective review of 65 patients with orbital pseudotumors treated at Massachusetts Eye and Ear Infirmary, Boston, USA, revealed isolated dacryoadenitis (n = 21), isolated myositis (n = 19), concurrent dacryoadenitis and myositis (n = 5), orbital apex syndrome (n = 6), and idiopathic inflammation involving the preseptal region, supraorbital region, sclera, Tenon capsule, orbital fat or optic nerve (n = 14). The mean age at presentation was 45 years. Pain and periorbital swelling were the most common clinical features and were observed in 45 (69%) and 49 (75%) patients respectively. Seventeen patients (26%) had bilateral involvement. Patients were treated with steroids alone (n = 45), steroids and subsequent radiation therapy (n = 8), steroids and nonsteroidal anti-inflammatory agents (n = 6), nonsteroidal anti-inflammatory agents alone in mild cases (n = 2), and rarely, radiation therapy without steroids (n = 1) or surgical debulking alone (n = 1).^35^

## Orbital Myositis

**Figure 5:**
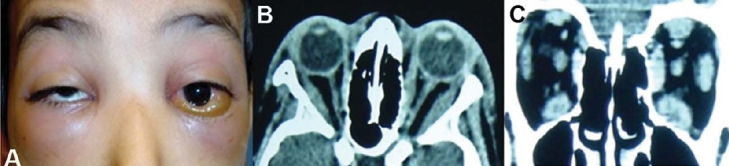
A 10 year old boy presented with fever, headache, !ì vision, chemosis, proptosis, restricted ocular motility anddecreased vision of both eyes (A). A CT-scan (axial and cronal) reveled bilateral extraocular muscle thickening involvingtendons (B and C).

**Figure 6:**
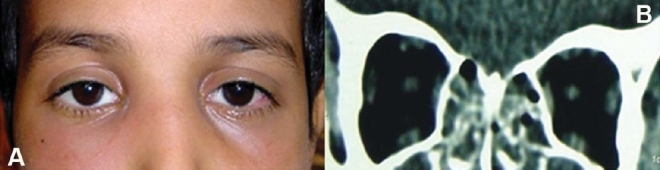
Patient (figure 5) showed dramatic improvement of his clinical symptoms and imaging studies aftertreatment with systemic corticosteroids (A and B).

Orbital myositis is a common component of orbital pseudotumor and 90-95% of cases are unilateral.^36^ Bilateral forms are exceptional and require search for specific etiologies particularly thyroid ophthalmopathy. Orbital myositis is considered as a subgroup of orbital pseudotumors primarily involving the extraocular muscles. The pathophysiology is still unknown. Patients typically present with orbital pain exacerbated by eye movement and diplopia (Fig 5). Response to steroids is dramatic (Fig 6).^37^ There is controversy about treatment options but corticosteroids are still the most common first choice therapy with good outcome.^38^,^39^ Orbital myositis occurs most frequently in young to middle-aged adults with a 2 to 1 female predominance. Other common findings include diplopia but minimal proptosis, conjunctival injection and chemosis and periorbital edema. Thyroid eye disease is commonly confused with orbital myositis, but the latter is characterized by a more acute onset, more severe pain and a rapid response to systemic corticosteroids therapy. Echography and CT-scan reveal enlarged muscle bellies and thickened tendons with low internal reflectivity.^23^ Although orbital myositis is generally considered to be an idiopathic inflammation, in certain patients it may be a manifestation of Lyme disease.^23^ Although the cause of orbital myositis is unknown, an immune-mediated pathophysiologic mechanism is likely.^40^ Kargi et al., 2005 reported a patient who was diagnosed with idiopathic orbital myositis based on the findings of diplopia, globe retraction on adduction and injection at the lateral muscle tendon insertion.^40^ Although orbital myositis as a cause of acquired retraction of the eye is rare, Kargi et al., 2005 emphasized the importance of globe retraction with injection over the recti as an important clue for the diagnosis of orbital myositis.^40^ Hattori et al., 2005 reported a case of 9-month-old girl who developed subacute orbital myositis presenting as limited adduction associated with blepharoptosis.^41^ An orbital MRI revealed swelling of the lateral rectus muscle with increased intensity on T2-weighted images with fat saturation which was enhanced with gadolinium.^41^ Attarian et al., 2003 reported three cases with acute orbital pain exacerbated on eye movement, enlargement of extraocular muscles on CT-scan with rapid response to immunomodulator treatment.^42^

The distinction of orbital myositis from Graves' ophthalmopathy is made on clinical grounds, with acute orbital pain and corticosteroids responsiveness being uncharacteristic of the latter. The possibility of orbital myositis being an immune-mediated process has been proposed.^43^ The role of ocular muscle biopsy is probably limited to atypical cases or to those unresponsive to steroid therapy, particularly to exclude neoplasia. Orbital myositis may be acute, subacute or recurrent. The acute form responds well to high doses of oral corticosteroids tapered gradually, but it may recur or become chronic. Extraocular muscle biopsy discloses features of non-specific muscle inflammation and no evidence of vasculitis. The subacute form of the disease responds less well.^44^ The combination of initial muscular inflammation and enlargement, subsequent fibrosis and restrictive extraocular motility and histologic evidence of these changes creates a useful model of orbital myositis. In one study a single injection of TPA (12-0-tetradecanoyl-phorbol-13-acetate) into the superior rectus muscle of New Zealand white rabbits produced inflammation and edema followed by fibrosis and muscle restriction. Clinical and histologic characteristics paralleled those of human idiopathic orbital myositis and thyroid orbital myopathy.^45^

In orbital myositis there may be isolated or multiple muscle involvement. A retrospective study of 100 patients with orbital myositis revealed that females were affected more than twice as often as males. Fifty-one patients (68%) had single muscle involvement with the lateral and medial recti affected most frequently. In 34 patients (45%), affected muscles functioned normally; the remaining 55% (63 muscles) were fairly equally distributed between paretic (20%), restrictive (20%) or combined paretic and restrictive (15%) myopathies. Fifty-one patients (68%) responded well to systemic corticosteroids, although 11 patients (15%) had further recurrences of the disease. Seven patients (9%) later developed thyroid eye disease after initially having unimuscular orbital myositis. Early institution of corticosteroids in order to avoid permanent restrictive myopathies have been advocated.^31^ Some cases of orbital pseudotumor may be related to preexisting vascular anomalies or orbital phlebitis. Two young female patients with acute orbital myositis associated with orbital hemorrhage and eyelid ecchymosis have been reported.^46^ These patients had painful proptosis, diplopia and CT-scan evidence of single extraocular muscle involvement with spillover of inflammatory edema into the adjacent orbital fat. Both patients were treated successfully with high-dose systemic corticosteroids.^46^ Orbital pseudotumor may present as a myositis isolated to the superior oblique muscle. Isolated involvement of the superior oblique muscle in association with orbital myositis in a 57-year-old man with acquired strabismus which was confirmed by CT-scan has been reported.^47^ Clinical and radiographic abnormalities quickly improved after oral steroid therapy.^47^ Oblique muscle involvement is infrequently reported, possibly because such involvement is difficult to identify clinically or by CT-scan. The oblique muscles may be frequently involved in orbital myositis when evaluated by U/S. In these cases U/S provides rapid, accurate, and reliable confirmation of the diagnosis of myositis, differentiates other orbital inflammatory diseases and provides an objective measure of the therapeutic response. Wan et al., 1988 reviewed seven cases of orbital myositis involving the oblique muscles solely or in association with rectus muscle.^48^ Echography demonstrated homogeneous low-reflective enlargement, diagnostic of myositis of the superior and inferior oblique muscles and tendons along their courses. In their study, findings of massive inferior chemosis was associated with involvement of the inferior oblique muscle.^48^

## Central Nervous System

Orbital pseudotumor with intracranial extension is rare. The principal symptoms include decreased vision and proptosis. Extension commonly develops through the superior orbital fissure into the middle cranial fossa and the cavernous sinus. It consists of a nonspecific infiltrate of the fatty tissue of the orbit that extends through one or more foramina into the adjacent intracranial compartment. The lesion mimics an infectious or neoplastic process. The initial treatment is a regimen of high-dose corticosteroids with radiotherapy given in unresponsive cases.^49^ A case of idiopathic hypertrophic cranial pachymeningitis associated with an orbital pseudotumor and granulocytic thyroiditis of unknown origin has been reported. The patient suffered from progressive loss of vision, as well as multiple cranial nerve dysfunctions. Medical treatment and radiation therapy were not beneficial.^50^ Orbital pseudotumor is a cause of secondary angle-closure glaucoma. Pathophysiology of angle-closure glaucoma secondary to orbital pseudotumor has been described using MRI and ultrasound biomicroscopy, which suggest angle closure from anterior rotation of the ciliary body due to choroidal effusions. In one of the study, two of the three cases resolved after treatment for orbital pseudotumor.^51^ Transient ischemic attack is rarely found in association with orbital disease and indicates possible intracranial extension. A patient with orbital pseudotumor established by biopsy developed episodes of transient sensorimotor hemiparesis. Neuroimaging showed intracranial extension of the disease with pronounced narrowing of the internal carotid artery in its intracavernous portion.^52^ Cyclophosphamide may produce a dramatic clinical response with marked radiological improvement that may be maintained for a long time.^53^

Orbital myositis cases with only neurological symptoms and without typical visual impairment are infrequent. A 24-year old patient with acute diplopia and headache without clinical manifestation on examination was diagnosed to have orbital myositis based on MRI and U/S.^54^ Isolated optic nerve involvement by orbital pseudotumor in patients who present with gradually progressive unilateral loss of vision may show dramatic response to a trial of corticosteroids.^55^ A case of bilateral infraorbital nerve involvement in orbital myositis has been described by CT-scan of the orbits showing enlargement of extraocular muscles. Biopsies of the inferior oblique muscle and infraorbital nerve showed the same inflammatory infiltrate: a mixed population of lymphocytes (B and T) with B cells expressing kappa and lambda light chains.^56^ Multifocal fibrosclerosis with CNS involvement have been described. Gilliard et al., 2000, described a case of cervical epidural pseudotumor in a 45-year-old man suffering from progressive quadriplegia who had an orbital pseudotumor 5 years earlier.^57^ In suspected cases of endocranial extension, CT-scan and MRI may help to diagnose orbital pseudotumor and demonstrate the presence of intracranial extension of orbital process. Orbital pseudotumor should be considered as a possible differential diagnosis when radiological studies reveal lesions extending from the orbit to intracranial structures.^58^

## Systemic Involvement

Orbital pseudotumor has been associated with ocular and systemic disorders, including scleritis, rheumatoid arthritis, Crohn's disease and systemic lupus erythematosis.^2^,^3^,^8^ Yan and Wu, 2002 studied the incidence of sinusitis in patients with orbital pseudotumor and speculated that sinusitis may be a cause in the etiology of orbital pseudotumor.^59^ Among the 209 patients with orbital pseudotumor, 36 (17.2%) had evidence of sinusitis which included 21 cases of maxillary, 15 case of ethmoid, 3 case of frontal and 3 case of sphenoid sinusitis. Orbital pseudotumor has systemic manifestations and may mimic more serious conditions, such as metastases from rhabdomyosarcoma or Ewing sarcoma, chronic recurrent multifocal osteomyelitis (CRMO), and SAPHO (synovitis, acne, pustulosis, hyperostosis, and osteitis). Imaging studies may show abnormalities in the parietal and frontal bones and distal right tibia. In one case orbital and tibial biopsies showed a nonspecific chronic inflammation in a 9-year-old girl with a 2-month history of the left orbit swelling and radiological evidence of right tibia abnormality.^18^ Giant cell myocarditis is a rare idiopathic inflammatory heart disease characterized histologically by multinucleated giant cells and clinically by rapid progressive heart failure, arrhythmias, or sudden death, often within hours to days of initial symptoms. Cases of giant cell myocarditis with orbital myositis have been reported. A case of a patient who had vitiligo, a diagnostic endomyocardial biopsy, survived because of a cardiac transplant. Giant cell myocarditis should be monitored in the course of orbital myopathy because of its life-threatening fulminant course.^60^ In one case, a 65-year-old woman developed progressive, bilateral ophthalmoplegia with thickened extraocular muscles on CT-scan. One month later, a cardiac arrhythmia led to her death. On histopathology, the extraocular and skeletal muscles showed diffuse mononuclear cell inflammation, while the heart contained granulomatous myositis. A patient's syndrome of orbital myositis and giant cell myocarditis may be a distinct nosologic entity.^61^ Patient may present with gradual and progressive visual loss and subsequent hypertension and pedal edema. Levine et al., 1993 reported a case of a 56-year-old man in whom a CT-scan of the orbits showed bilateral diffuse retrobulbar masses and an abdominal CT-scan reveled a diffuse retroperitoneal mass invading the aorta, ureters and inferior vena cava.^62^ Biopsies of the orbit and abdominal masses confirmed a heterogeneous cell population and marked fibrosis consistent with a sclerosing inflammatory process. Bilateral sclerosing orbital pseudotumor should cue the physician to suspect coexisting systemic disease.^62^ Orbital myositis with atypical features of acute-on-chronic course, and concomitant sinus disease have been reported.^63^ A 70-year-old man, who had been diagnosed with sclerosing mesenteritis following an abdominal biopsy, presented with an acute onset of left upper eyelid swelling, moderate ptosis, mild chemosis and restriction of movements. A CT-scan showed an enlarged lateral rectus muscle with surrounding soft tissue changes. A diagnosis of orbital myositis was made and the patient was commenced on treatment.^22^

## Histopathology

**Figure 7:**
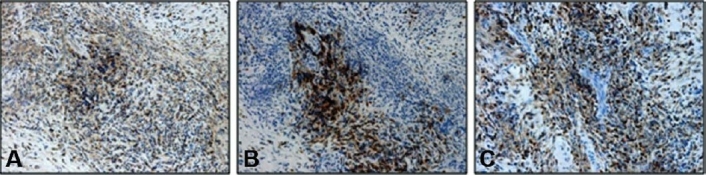
Immunohistochemistory performed on a biopsy specimen obtained from patient in figure 2 reveling stainingfor T-lymphocytes using CD3 antibody, X20 (A), for B-lymphocytes using CD20 antibody, X20 (B) and for T-lympho-cytes using CD45 antibody, X20 (C).

**Figure 8:**
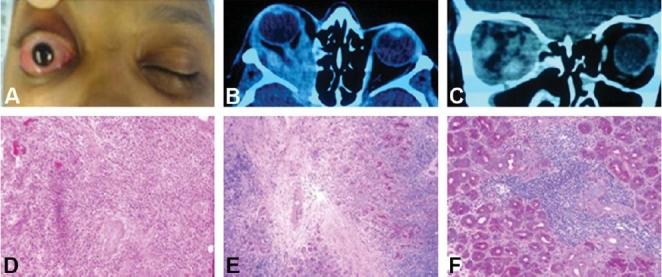
A 45-year-old woman with significant loss of vision in her right eye despite aggressive treatment withcorticosteroids (A). CT-scan (axial and coronal) demonstrated thickening of all recti muscles and proptosis onthe right side (B and C). On histopathology, there is evidence of chronic inflammation (D), sclerosing fibrosis(E) and chronic dacryoadenitis (F).

Pseudotumor of the orbit comprises of nonspecific polymorphic, lymphocytic infiltrates with macrophages, polymorphonuclear leukocytes and eosinophilis.^2^,^3^,^9^ Increased connective tissue with edema and fibrosis is also commonly seen (Fig 7). When extensive fibrosis formation is seen on the biopsy specimen, it is termed sclerosing orbital pseudotumor. The calcifying orbital pseudotumor is a very rare disorder due to a chronic, idiopathic inflammatory process of the orbit. Chronic granulomatous types may eventually transform into the sclerosing type (Fig 8). The granulomatous type shows a good response to corticosteroids as well as radiotherapy. In general, calcifying pseudotumors can be treated only by operative exploration and tumor removal. Endonasal approach can be used to access calcifying orbital pseudotumor of the orbital apex.^64^ Sclerosing orbital pseudotumor is a distinct form characterized by slow and relentless involvement of orbital structures with insidious course that makes distinction from neoplastic lesions clinically difficult.^27^,^65^ It has been proposed that when orbital pseudotumor presents as a bilaterally diffuse retrobulbar apical mass, the sclerosing subtype must be considered first and an orbital biopsy be performed. The most common ocular findings in patients with sclerosing orbital pseudotumor may be decreased vision, proptosis, and restriction of extraocular muscles.^66^ A recent multicenter review of the clinical features and treatment of 31 patients with sclerosing orbital pseudotumor including all patients with histologically confirmed cases from 5 regional orbital centers revealed that the average duration of symptoms at presentation was 13.4 months with predilection for the lateral and superior quadrants, 13 patients had apical disease, and 4 had extraorbital involvement.^33^ Histopathological findings invariably showed sclerosis associated with a sparse mixed cellular infiltrate. Of the 27 patients treated with oral Prednisolone, response to treatment was good in 9 patients, partial in 11, and poor in 7. Six patients were treated with a second-line immunosuppressive agent, and 6 received radiotherapy. The response to radiotherapy was generally poor. Sclerosing orbital pseudotumor is a rare condition that can be difficult to diagnose and manage. Early intervention with immunosuppression by corticosteroids combined with second-line agents can result in control and regression of the disease.^33^

Histologic similarity and clinical association of sclerosing orbital pseudotumor with other fibrosclerosing conditions has been documented.^67^ A case of orbital pseudotumor in conjunction with autoimmune hemolytic anemia in a 59-year-old woman with left upper lid swelling, periocular pain, proptosis and restricted ocular motility has been reported. Homogeneously enhancing lateral orbital mass was seen by MRI. Biopsy revealed dense fibrous connective tissue with a paucicellular infiltrate, consistent with orbital pseudotumor and treatment with Prednisolone 60 mg/day resulted in the resolution of proptosis and improved extraocular movements.^67^

## Pediatric Orbital Pseudotumor

In children, systemic signs may include headache, emesis, anorexia, lethargy and fever in upto 50% of the patients,^14^–^16^ which are rarely reported in adult patients. Other distinctive characteristics of pediatric orbital pseudotumor may include, bilaterality in 45% of cases (Figure 5), iritis and peripheral eosinophilia.^16^ Some of the differential diagnosis of pediatric orbital pseudotumor may include orbital cellulites, rhabdomyosarcoma, leukemia, orbital trauma with retained foreign body, ruptured dermoid cyst, lymphangioma, neuroblastoma, metastatic retinoblastoma and thyroid related orbitopathy.^16^ Laboratory studies which may be of help include elevated erythrocyte sedimentation rate, peripheral blood eosinophilia and serum antinuclear antibody (ANA) test in the setting of Tolosa-Hunt suspect.^2^,^9^,^10^,^28^,^33^ Yan et al., 2006 studied clinical features of orbital pseudotumors in Chinese children.^68^ Ocular motility restriction (46%), swollen eyelid (42%), proptosis (42%) and high orbital pressure (42%) were the most common presenting signs in children with orbital pseudotumor. Ptosis occurred more often in pediatric orbital pseudotumor cases (38%) than in adult cases (9%). According to radiological and surgical findings, a local mass within the orbit was the most frequent subtype (50%), followed by dacryoadenitis (29%), myositis (8%), perineuritis (4%), eyelid pseudotumor (4%) and diffuse orbital inflammation (4%). In their study pediatric orbital pseudotumor accounted for 11.5% of all patients.^68^ An unusual case of orbital pseudotumor presenting with bilateral exudative retinal detachment in a 9-year-old girl has been described. Prompt diagnosis and corticosteroid treatment resulted in good clinical response and significant visual recovery.^69^

## Treatment

Systemic corticosteroid therapy is the cornerstone of managing orbital pseudotumors. Over 75% of patients show dramatic improvement within 24 to 48 hours of treatment (Figure 2, 4 and 6).^2^,^3^,^8^ Improvement with corticosteroids therapy is of diagnostic significance specifically a corticosteroid responsive orbital process is more likely to correspond to pseudotumor.^8^ Other associated abnormal laboratory tests may also respond to systemic corticosteroid treatment. A case of idiopathic hypereosinophilic syndrome in a 53-year-old man associated with an orbital pseudotumor has been reported.^70^ Therapy with corticosteroids rapidly decreased the number of circulating eosinophiles; the orbital pseudotumor regressed at the same time.^70^ Two cases of orbital myositis with monoclonal gammapathy of undetermined significance have been reported.^71^ In the first case, the patient achieved remission with steroid pulse therapy followed by administration of high doses of a steroid. In the second case, because of repeated recurrences the patient was treated with steroid pulse therapy and then radiation therapy to achieve complete remission.^71^ In a study from Turkey, high-dose oral corticosteroid treatment was found to be successful in significant number of patient and radiotherapy was further helpful in those patients resistant to corticosteroids. One resistant case responded to Cyclophosphamide and 2 cases with focal mass lesions were treated with orbitotomy. Four additional cases had spontaneous remission. Overall almost 80% of patients eventually had a good outcome.^19^

Recurrences are common in orbital pseudotumor especially with bilateral disease process. Normally, starting dosages of Prednisone 1.0 to 2.0 mg/kg/day are adequate. When improvement is noted, dosages should be continued with a slow tapering guided by clinical judgment. Intraorbital injection of corticosteroid has been found to be useful and effective treatment of orbital pseudotumor and may be considered as a first-line treatment in selected patients.^72^ Clinical efficacy of curcumin, the active constituent of rhizomes of Curcuma longa, in the treatment of patients suffering from orbital pseudotumors have been reported.^73^ Oral administration at a dose of 375 mg/3 times/day for a period of 6-22 months in eight patients showed complete recovery in 4 without any side effects.^73^ Low-dose radiation is typically reserved for elderly patients or for those unresponsive to systemic corticosteroids or in whom steroids are contraindicated.^2^,^8^,^65^ Radiation therapy in doses of 20 Gy in 10 fractions for patients with orbital myositis appears to be effective in palliating symptoms, but long-term control is not satisfactory.^74^ For those patients who are refractory to both corticosteroids and radiotherapy, use of chemotherapeutic agents such as Cyclophosphamide, Methotrexate and Cyclosporine have been found to be helpful.^10^

Other therapies with promise include cytotoxic agents, (Cyclophosphamide and Chlorambucil), immunosuppressants (Methotrexater, Cyclosporine, Azathioprine), IV immunoglobulins, TNF-alpha inhibitor, monoclonal antibody (Infleximab and Adalimumab) and Mycophenolate Moftil which inhibit denovo purine synthesis and prevent B & T lymphocyte replication.^7^,^75^–^78^ Benefit of Cyclosporine treatment for orbital myositis have been demonstrated.^79^ Treatment with Infleximab (monoclonal antibody directed against TNF-alpha) appears to offer another therapeutic option in cases of recalcitrant or recurrent orbital pseudotumor in which conventional treatment fails. Favorable response to treatment with Infleximab in several patients with chronic and difficult-to-control orbital pseudotumor after the failure of traditional therapy, which included corticosteroids, radiotherapy or anti-inflammatory chemotherapeutic agents, has been reported. Pain, swelling, and decreased need for concomitant corticosteroids are the primary measures of treatment success. In one study, treatment with Infleximab helped in the improvement of comorbid disease symptoms (Crohn disease in 2, Behcet disease in 1 and psoriasis in 1). There were no untoward effects of treatment after a mean follow-up of 15.7 months (range 4 to 31 months).^76^ Mycophenolate Moftil therapy resulted in the resolution of inflammation in 4 patients with refractory or corticosteroid-dependent orbital pseudotumor and was used successfully for a first episode of orbital pseudotumor in a patient for whom corticosteroids were contraindicated.^77^ A patient in whom a poor initial response to oral steroids necessitated an orbital biopsy showing a paucicellular lymphocytic infiltrate with areas of fibrosis replacing normal tissue confirming sclerosing orbital pseudotumor has been reported. Subsequently the patient developed exposure keratopathy and diplopia associated with eyelid retraction, lagophthalmos, and decreased motility despite increasing doses of steroids and intralesional steroid injections. In one report CyberKnife radiosurgery and Rituximab resulted in symptomatic resolution, improved eyelid closure, motility and almost complete radiographic regression of his disease process which was maintained upto 18 months of follow-up period.^75^
